# “God gave you a special child because you are special”: difficulties, coping strategies, and parental burnout of Jewish mothers – a qualitative study

**DOI:** 10.3389/fpsyg.2023.1259670

**Published:** 2023-12-05

**Authors:** Yifat Findling, Sivia Barnoy, Michal Itzhaki

**Affiliations:** Nursing Department, School of Health Professions, Faculty of Medicine, Tel Aviv University, Tel Aviv, Israel

**Keywords:** parental burnout, mothers of children with special needs, ultra-orthodox, caregiver burden, coping strategies, emotional coping, spiritual coping, social support

## Abstract

**Background:**

“Parental Burnout” is a specific syndrome resulting from an imbalance between continuous exposure to chronic parenting stress and available protective resources. Mothers of children with special needs have an increased risk of Parental Burnout due to dealing with various difficulties from carrying out long-term childcare.

**Purpose:**

To examine difficulties, coping strategies, and PB (risk factors and protective resources), among ultra-Orthodox and non-Orthodox Jewish mothers of children with special needs with high or low levels of Parental Burnout.

**Methods:**

A qualitative study. Twelve mothers of children with special needs identified with high or low Parental Burnout via a quantitative study were interviewed. The constant-comparative analysis method was used to analyze the findings.

**Findings:**

Three themes and twelve categories emerged: 1. Difficulties involved in motherhood with six categories: (a) caregiver burden, (b) task overload, (c) physical and mental self-neglect, (d) environmental, couple-hood, family and social neglect, (e) recognizing the gap between their child with special needs and other children of the same age, (f) the environment’s contradictory expectations from the mother; 2. Coping strategies with four categories: (a) practical, (b) spiritual, (c) social and (d) emotional; and 3. Parental Burnout, consequences and coping with two categories: (a) personal and environmental risk factors (e.g., fear of the future, difficulty in trusting others in taking care of their child), and (b) personal and environmental protective resources (e.g., sharing similar circumstances with other mothers, a belief in God).

**Conclusions and implications:**

The findings contribute to understanding the unique difficulties, coping strategies and differences in risk factors and protective resources of Parental Burnout among ultra-Orthodox and non-Orthodox Jewish mothers of children with special needs. In order to alleviate the difficulties experienced by mothers and thus also reduce Parental Burnout risk factors and provide effective protective resources, it is recommended to develop empirically based professional guidance for policymakers, child development specialists, nurses, physiotherapists, and informal caregivers.

## Introduction

Parenting is one of the most rewarding and challenging experiences in a person’s life. It usually contains moments of happiness, joy and pleasure, but it may also be accompanied by stressful situations, feelings of exhaustion and ongoing stress due to continuous responsibilities (Nelson et al., 2014; Nomaguchi and Milkie, 2020). Despite the growing social recognition of the importance of shared parenting and equal parental responsibility, mothers are still considered to be the primary informal caregivers of their children; therefore, they are more likely to experience mental and physical exhaustion than their spouses (Bristow et al., 2018; Masefield et al., 2020). Parenting children with special needs (W-SND) with an ongoing chronic disability requires long-term care. The need to cope with the burden of care for a long time involves many pressures (such as physical, emotional, economic, and social) and might trigger high levels of stress in parents, placing them at increased risk of Parental Burnout (PB) and impaired quality of life (Carroll, 2013; Liu et al., 2020).

PB is defined as a specific burnout syndrome that is related to and arises from parental roles. It mainly develops due to an imbalance between burnout risk factors (continuous exposure to ongoing demands such as irregular and unusual pressures, difficulties, and family stress), and protective factors against burnout (the availability and quantity of coping resources) (Mikolajczak et al., 2018a,b). PB includes physical and emotional exhaustion, emotional distancing from the child, saturation from the parental role, and a contrast with the previous parental self (Mikolajczak et al., 2018a,b). As a result of PB, parenting might become functional where parents’ interaction with their children is limited to only the practical aspects (Mikolajczak et al., 2018a,b; Van Bakel et al., 2018).

The factors that may affect PB are related to the parents’ personality, their family relationships and functioning, as well as socio-demographic and socio-cultural characteristics. The *personal* risk factors include lack of emotional control (Le Vigouroux and Scola, 2018), perfectionism (Kawamoto et al., 2018; Lebert-Charron et al., 2018), neuroticism (emotional imbalance and difficulty managing emotions) (Gérain and Zech, 2018; Lebert-Charron et al., 2022), and depression (Roskam et al., 2017; Lebert-Charron et al., 2018). Additionally, low emotional intelligence (Mikolajczak et al., 2018a,b), low self-esteem, a high need for parental control (Lindström et al., 2011), parental strictness and maternal emotional exhaustion (Roskam et al., 2017). Risk factors related to the *family* include a dysfunctional family circle, especially dissatisfaction with conjugal relationships and lack of partner support (Gérain and Zech, 2018; Le Vigouroux and Scola, 2018; Lebert-Charron et al., 2022). Also, role restriction in parenthood and attachment avoidance (Mikolajczak et al., 2018a,b). Among the *socio-demographic* factors, having young children under five years was found to be a significant risk factor for PB (Lindström et al., 2011; Mikolajczak et al., 2018a,b).

However, there are possible available protective resources, which can significantly reduce parental stress and alleviate burnout. These include *personal* factors, such as high emotional intelligence, high self-efficacy, high self-compassion, positive thinking, and proper management of leisure time. Protective factors related to the *family* include high parental skills, positive parenting, co-parented agreement, a coherent family environment, and marital satisfaction (Le Vigouroux et al., 2017; Mikolajczak et al., 2018a,b). Moreover, agreeableness (friendly and empathic behavior) and perseverance, are characteristics that allow parents to identify their children’s needs better and respond to them efficiently (Le Vigouroux and Scola, 2018). From a *sociocultural* point of view, support from the environment and society is an important protective resource (Le Vigouroux and Scola, 2018; Mikolajczak et al., 2018a,b).

To date, most studies have focused on PB among parents of healthy children, with only a few studies conducted specifically among parents of children with chronic diseases (Lindström et al., 2010; Vinayak and Dhanoa, 2017; Gérain and Zech, 2018). Also, few studies have examined sociocultural aspects related to PB among parents of children W-SND generally and mothers specifically. Thus, to examine the possible socio-cultural effect on PB, both ultra-Orthodox and non-Orthodox Jewish mothers were interviewed. In Israel, the ultra-Orthodox society is considered a conservative religious community with a lifestyle led according to Jewish law precepts. These affect many aspects of life, such as wardrobe, nutrition, education, health, and family life. This society is characterized by a separation from the general population and a tendency toward communal life in which the family and community support the individual when needed (Taub and Werner, 2016; Kalaji and Brown-Levinson, 2017). The prevailing ultra-Orthodox view is that the birth of a child with a disability stems from God’s will, who will also provide them with tools to cope with the situation. They believe that each child is born to fulfill a certain role in the world and therefore the child needs help to achieve this role (Kandel, 2010). At the same time, the attitude of the ultra-Orthodox family and community environment toward persons with special needs and disabilities in the family is ambivalent: moral responsibility and kindness as opposed to an attitude of shame and rejection toward the “different” due to a fear of harming their social status in the community (Shaked and Bilu, 2006; Kandel, 2010; Ivry et al., 2011). The aim of this study was to examine in depth the experiences of ultra-Orthodox and non-Orthodox Jewish mothers of children W-SND with high or low levels of PB.

## Materials and methods

### Study procedure

The study used a phenomenological approach to construct a grounded theory (Glaser and Strauss, 1967; Creswell and Poth, 2018). This qualitative study is part of a mixed-methods research which included a quantitative phase (questionnaires) followed by a qualitative phase (interviews). The qualitative study aimed to enable in-depth understanding of the unique difficulties, coping strategies and PB factors and consequences among non-Orthodox and ultra-Orthodox Jewish mothers of children W-SND with high or low levels of PB.

The quantitative study examined the level of PB of 176 mothers of children W-SND (compared to 176 mothers of children WO-SN), and their background data were collected after conducting a pilot study to validate the research system and tools (Findling et al., 2022). The results of the first quantitative phase of the research indicated that PB of mothers of children W-SND (non-orthodox and ultra-orthodox) was significantly higher than that of mothers of children WO-SN. Following these results, in order to identify the mothers’ level of PB, the risk levels were calculated according to the suggestion of Roskam et al.’s “A step forward in the conceptualization and measurement of Parental Burnout,” (Roskam et al., 2018) as follows: the sum of the responses (range 23–161 [23 × 1 through 23 × 7]) to all the items was categorized into a five-rank scale of risk of PB: 0 = no PB (< 35), 1 = low risk (Tix and Frazier, 1998; Tarakeshwar and Pargament, 2001; Green, 2007; Broberg, 2011; Neely-Barnes et al., 2011; Pisula, 2011; Pillay et al., 2012; Ahles et al., 2016; Gonen, 2016; Singal, 2016; Tsai et al., 2016; Vanegas and Abdelrahim, 2016; Ahmed and Al-Mosawi, 2018; Fusch et al., 2018; Mitter et al., 2019; Noble and Heale, 2019; Enea and Rusu, 2020; Tsibidaki, 2022), 2 = medium risk (54–70), 3 = high risk (71–88), and 4 = has PB (89+). Following this classification, 22 mothers of children W-SND who agreed to be interviewed in the quantitative questionnaire, were invited to participate in the study [12 with low risk for PB (Nelson et al., 2014)] and ten with high risk (Bristow et al., 2018) or have PB (Masefield et al., 2020). Twelve mothers, out of these 22 mothers, expressed agreement to be interviewed in the qualitative phase of the study.

### Participants and sample

Twelve mothers of children W-SND took part in this study: six of them with high PB (three from the ultra-Orthodox sector and three from the non- Orthodox sector) and six with low PB (three from the ultra-Orthodox sector and three from the non- Orthodox sector) see Table 1. According to the literature, the minimum sample size to achieve saturation in qualitative data analysis is at least 12 respondents (Fugard and Potts, 2015; Braun and Clarke, 2019; Kegler et al., 2019). The mean age of mothers with high PB was 41.2 years (SD = 2.3) and of mothers with low PB was 40.3 years (SD = 7.5). Most of them were married (83%, *n* = 10) with an academic education (mothers with high BP: 83%, *n* = 5, with low BP: 66.7%, *n* = 4). Half of them worked part-time, one-third full-time, and two (with high/low burnout) were not working.

**Table 1:** Sociodemographic characteristics of the mothers (*n* = 12).

PB	Low (*N* = 6)	High (*N* = 6)
	Mean (SD)	Mean (SD)
*Mother’s age (years)*	40.3 (7.5)	41.2 (2.3)
*Special needs child’s age (years)*	4.3 (0.6)	4.1 (1.1)
*Number of children in the family*	4.5 (3.0)	3.5 (1.5)
*Sector*	Number (%)	Number (%)
Ultra-Orthodox	3 (50.0%)	3 (50.0%)
Non-Orthodox	3 (50.0%)	3 (50.0%)
*Marital status*		
Married	5 (83.3%)	5 (83.3%)
Single parent	1 (16.7%)	0 (0%)
Separated	0 (0%)	1 (16.7%)
*Education*		
High school	2 (33.3%)	1 (16.7%)
Academic	4 (66.7%)	5 (83.3%)
*Job scope*		
Full time	2 (33.3%)	2 (33.3%)
Part-time	3 (50.0%)	3 (50.0%)
Not working	1 (16.7%)	1 (16.7%)
*Order among siblings*		
1st	1 (16.7%)	1 (16.7%)
2nd	1 (16.7%)	4 (66.7%)
3rd	2 (33.3%)	0 (0%)
5th	1 (16.7%)	0 (0%)
6th	0 (0%)	1 (16.7%)
10th	1 (16.7%)	0 (0%)
*Childs’ sex*		
*Boy*	4 (66.7%)	5 (83.3%)
*Girl*	2 (33.3%)	1 (16.7%)
*Type of special needs/disability/impairment*		
Communicational	3 (50.0%)	3 (50.0%)
Physical	1 (16.7%)	0 (0%)
Developmental	1 (16.7%)	0 (0%)
Mental development	1 (16.7%)	0 (0%)
Physical mental development	0 (0%)	2 (33.3%)
Developmental, language, developmental	0 (0%)	1 (16.7%)

Children W-SND in this study had an ongoing chronic disability (over a year) and were treated in a child developmental center, according to a physician’s diagnosis. The sample included children with the following disabilities: communication – 44.9%, physical – 30.1%, cognitive developmental – 27.8%, visual sensory – 6.8%, auditory sensor – 5.1%, and mental – 2.8%. The children’s age range was two and a half to five years, and most of them were boys (mothers with high BP: 83.3%, *n* = 5, with low BP: 66.7%, *n* = 4). The mean age of children of mothers with high PB was 4.1 years (SD = 1.1) and of children of mothers with low PB was 4.3 years (SD = 0.6). Overall, on average, families of mothers with high PB had fewer children (*M* = 3.5, SD = 1.5) than mothers with low PB (*M* = 4.5, SD = 3.0) (Table 1).

### Data collection

The data were collected using a semi-structured interview guide constructed by the researchers during January–March 2022. The first author interviewed the mothers remotely via Zoom due to a lockdown during a COVID-19 peak. The duration of each interview was between 1 h and an hour and a half. The mothers were asked to describe experiences, feelings, thoughts, and behaviors related to being mothers of children W-SND. The interview guide included the following questions: Tell me about your child W-SND; Are you experiencing difficulties as a mother of a child W-SND? If so, what are they? Which emotions do you feel as a mother of a child W-SND? What do you think society expects you to feel as a mother of a child W-SND? They were also asked about the factors they thought might contribute to the development of PB: How willing are you to accept help from others to care for your child? To what extent do you initiate getting help from others for caring for your child? To what extent do you feel burned out as a mother of a child W-SND? What are the factors that can contribute to PB, in your opinion? What are the factors that may protect mothers of children W-SND from PB, in your opinion? Do you have any insights, tools, or tips for coping with PB that you can recommend to mothers who have a child W-SND?

### Data analysis and trustworthiness

The answers were recorded (with the mothers’ consent) and transcribed by the first author qualified in advanced qualitative research methods; then, the entire transcriptions of the interviews were read twice. The transcribed interviews were formatted as a table, which included the identification of the mothers, the questions according to the interview guide, the mothers’ answers divided into *“*units of meaning*”* (from one word to a sentence or paragraph that expresses a single idea) and comments identified by labels. The grounded theory was constructed by labeling repeated data and comparing codes and categories, i.e., the constant comparative analysis procedure (Glaser and Strauss, 1967).

Following this, the first author identified and labeled repeated findings, coded and categorized them into similar contents, and divided them into themes, groups of themes, and main categories (Fram, 2013). The third author, an expert on qualitative research methods analyzed the data independently. The combined findings were approved by the second author, who has extensive experience in conducting mixed-methods research. The three authors conducted a peer debriefing procedure to ensure the reliability and validity of the thematic construct of the findings (Lincoln and Guba, 1985). The thematic construct was also presented to two mothers who participated in the interviews (selected randomly). The three researchers discussed the classification of themes and categories until full agreement and saturation were reached, according to the Comparative Method for Themes Saturation (CoMeTS) (Constantinou et al., 2017). Both the emic perspective of the mothers (inner view) and the etic theoretical perspective (external concepts) were combined for naturalistic inquiry (Fram, 2013). For each quote, the mother’s age, sector, and level of PB are presented in brackets to maintain their anonymity (see Results section). This procedure was carried out according to the Consolidated criteria for reporting qualitative research (COREQ, Tong et al., 2007).

### Study rigor

The validity of the qualitative findings was ensured following the guidelines of Lincoln and Guba (1985), as follows: Credibility – internal validity was confirmed using a phenomenological approach to construct a theory grounded in the field. Transferability – an elaborated description of the ultra-Orthodox and non-Orthodox Jewish mothers of children W-SND samples was provided. The dependability of the data over time should be tested via further research, as this was a preliminary study of PB among mothers of children W-SND. Confirmability – all findings were based on quotes from the mothers’ answers. Authenticity – the interviews were conducted remotely via Zoom due to lockdowns. Nevertheless, it is recommended to conduct them in the familial natural environment, as was planned initially before the COVID-19 pandemic.

Triangulation was carried out through three methods: (i) methodological triangulation – the research design was a mixed methods design (first a quantitative survey followed by qualitative semi-structured interviews); (ii) data triangulation – data were gathered twice (through the questionnaire and later from the interviews) in different spaces (questionnaires were filled out in the centers and the interviews were carried out remotely via Zoom); and (iii) investigator triangulation – the qualitative data were analyzed independently by the two researchers (the first and third authors) and validated by the second author (Fusch et al., 2018; Noble and Heale, 2019).

### Ethical consideration

The study was approved by the Helsinki Committee of Meuhedet Health Service Clinics (Approval number 02290120) and by Tel-Aviv University’s Ethics Committee (Approval number 0001222-2). The researcher explained to the mothers the purpose of the interview and the importance of their participation in the study. They were also assured that they could terminate their participation in the interview at any time, without the need for explanation, and that their identities would be kept confidential. The interviews were recorded and transcribed following the mothers’ consent.

## Results

**Figure 1:**
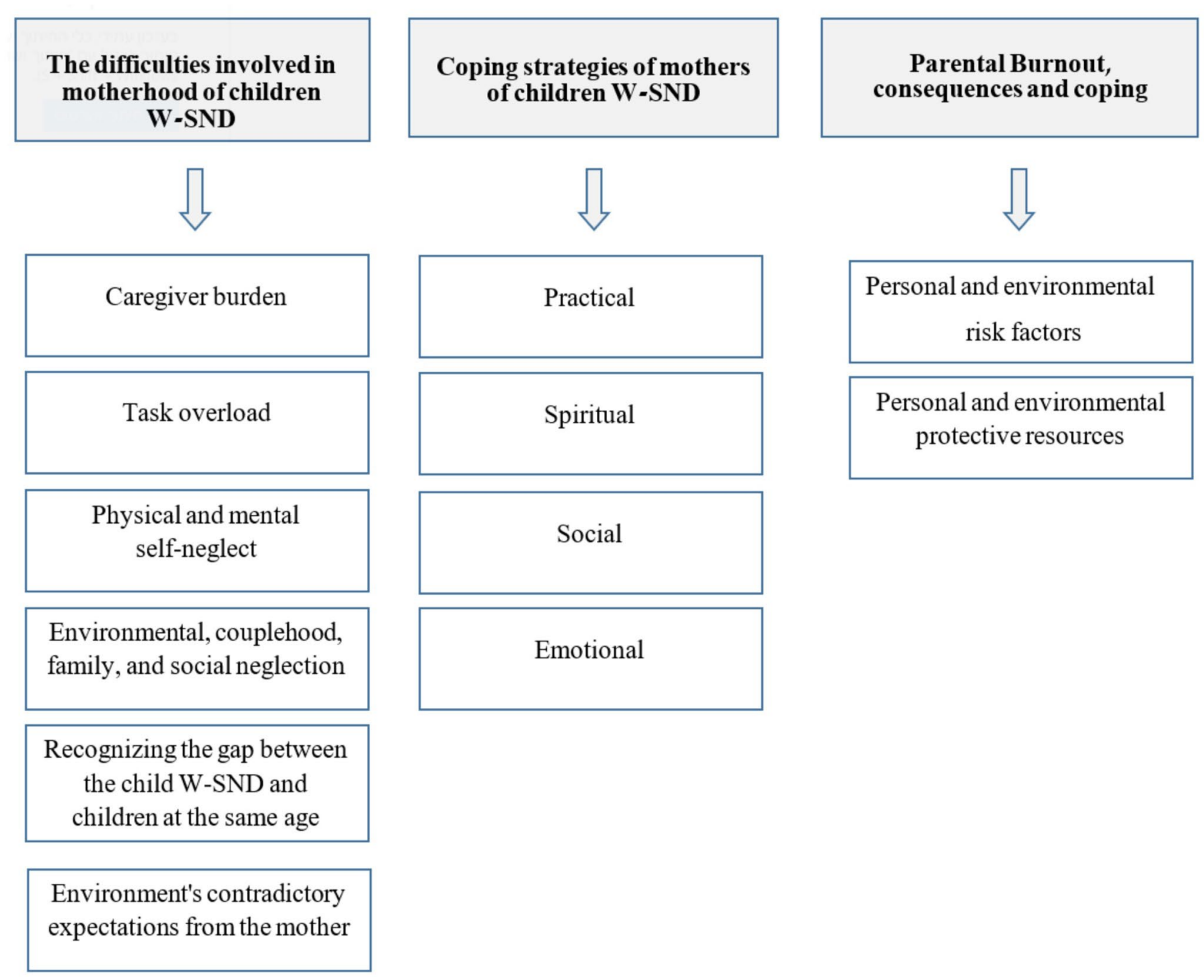
The research themes and categories.

The themes and categories that emerged from the qualitative analysis are presented in Figure 1.

The thematic analysis revealed three themes and 12 categories: 1. Difficulties involved in motherhood: (a) caregiver burden, (b) task overload, (c) physical and mental self-neglect, (d) environmental, couple-hood, family and social neglect, (e) recognizing the gap between the child with special needs and other children of the same age, (f) environment’s contradictory expectations from the mother; 2. Coping strategies: (a) practical, (b) spiritual, (c) social, and (d) emotional; and 3. Parental Burnout, consequences and coping: (a) personal and environmental risk factors, and (b) personal and environmental protective resources.

### First theme. Difficulties involved in the motherhood of children W-SND

The first theme dealt with the difficulties involved in the motherhood of a child W-SND (caregiver burden, task load, self-neglect and social neglect, recognition of the gap between the child W-SND and other children of the same age, and the environment’s contradictory expectations from the mother regarding their emotions and thoughts).

#### Category 1. Caregiver burden

According to all mothers, there are difficulties surrounding their child’s care; it requires a lot of time, attention and supervision of the child according to their functional condition:

- “There is a lot of overload including physiotherapy, occupational therapy, and speech therapist. There is also a fear that something dangerous will happen to him and that he must be guarded at all times” (39, non-Orthodox, high burnout).
- “It was hard for me in the midst of all the tasks to look after him and my other children. It’s also not easy with the child’s treatments twice a week, riding the bus with him, and I have just given birth” (31, ultra-Orthodox, low burnout).

The mothers also described difficulty coordinating the many treatments for the child, especially when they saw themselves as the primary caregivers of the child:

- “The entire therapeutic burden is on me. It’s also very difficult for me vis-à-vis the bureaucracy to approve all sorts of things related to childcare, treatments, allowances, therapeutic frameworks” (42, ultra-Orthodox, high burnout).

#### Category 2. Task overload

All mothers reported overload resulting from the multitasking they had to deal with in addition to the tasks related to caring for their child. These included the need to take care of their other children, spend time with them, keep the house functioning, clean and tidy, and have time to cook and bake.

- “There’s a crazy excess of tasks. I have other children to take care of, my partner does not help me at all” (42, ultra-Orthodox, high burnout).
- “I have more children and more tasks, and I do not have time left for myself, for my health and the other children, the load was mainly related to caring for the child and at the same time also at home, meaning getting everything done cooking, baking, reaching places that are not reached while caring for the child” (31, ultra-Orthodox, low burnout).

#### Category 3. Physical and mental self-neglect

The mothers said they did not eat well, lost weight, and suffered from fatigue and chronic deprivation of sleep. Some noted that they were in untreated states of depression and anxiety. Mothers with high burnout described this as an ongoing condition that had not yet been treated:

- “I was very miserable, really depressed, I wasn’t in the mood, I ate disorderly, I did not sleep, I did not leave the house except for what was necessary, and I really neglected my mental state and never went to receive treatment” (43, ultra-Orthodox, high burnout).
- “All the thoughts about the difficulties I have is reflected in my health and until now I have really neglected my mental health and have not sought treatment” (38, non-Orthodox, high burnout).

However, mothers with low burnout reported that this condition was related to certain time periods, especially in the time just after their child had been diagnosed, and some even noted that over time there was an improvement and they had less self-neglect:

- “There were times, especially in the beginning after diagnosis, when I really neglected myself and my health, I lost a lot of weight and I had no desire to eat or leave the house, it was really such a deep depression. I was constantly focused on the child’s treatments” (34, non-Orthodox, low burnout).
- “In the first year, everything came at the expense of other things, there was no mother at home. There was only me, the mother of D (the child with special needs). I neglected everyone, myself and my mental health. Today I’m in a different place, making time for everything, for me, for the children” (37, ultra-Orthodox, low burnout).

#### Category 4. Environmental, couple-hood, family, and social neglect

Caring for a child W-SND mainly affected the lack of quality time with the mothers’ other children and their spouse and they reported difficulty in being able to socialize:

- “The treatment also comes at the expense of the relationship, my personal time and spending time with my older daughters. I used to spend every night with them before bed and now that’s not happening. I used to go out with my husband a lot and now we almost do not. My friends were pushed aside. There are no cafes, no entertainment, nothing. It’s all about caring for the child” (41, non-Orthodox, high burnout).
- “Caring for him (child W-SND) comes at the expense of many things, quality time with my husband, I want to pamper the other children and cannot find time for it” (44, ultra-Orthodox, high burnout).

Mothers with low burnout noted that environmental neglect occurred mainly in the time immediately after their child’s diagnosis compared to the present:

- “There were times when I really neglected my relationship with my spouse. I also did not tell anyone about the situation except my mother and a few other people. Today it’s much better, I’m still concentrating on taking care of the girl, but because I see she’s very advanced, I also allow myself time for other things” (34, non-Orthodox, low burnout).

#### Category 5. Recognizing the gap between the child W-SND and other children of the same age

All mothers described the difficulty and pain they experience when they see their child’s development compared to other children of the same age whose development is normative, especially in social meetings:

- “There are very large developmental gaps and I feel great pain, especially in social events, seeing my child lagging behind other children his age in terms of development” (42, ultra-Orthodox, high burnout).
- “It’s very hard for me when I see the gap between him and children his age, even though his functioning is relatively high” (38, non-Orthodox, high burnout).
- “It’s very difficult for me when I compare him to other children his age. Now everyone is almost 4 years old, talking, active, independent and only my child is left behind. Suddenly the gap is reflected in front of my face, and it’s difficult” (37 ultra-Orthodox, low burnout).

#### Category 6. Environment’s contradictory expectations from the mother

Mothers, members of the community, family, co-workers, neighbors, and friends have conflicting expectations about how they should feel or function. On the one hand, they are expected to be strong mothers, behave normally, and continue to function, and on the other hand, they are expected to feel worried, anxious, and frustrated about their child’s condition. The mothers from the ultra-Orthodox sector mainly, described statements from their friends, neighbors, and people in their community that were difficult for them to hear and that aroused feelings of anger and pain:

- “People around me told me, ‘God gave you a special child because you are very special’, and my family and friends expect me to continue behaving normally and be happy and come to their celebrations. It is very painful and difficult for me; they expect me to be strong and not to break down” (42, ultra-Orthodox, high burnout).
- “People from the community expected me to be strong, to be happy with what I received, to be proud and grateful that as if I had received a gift from Heaven, some told me, you are a special woman, and you received a special child for a reason. They do not understand that no one asks for such gifts” (37, ultra-Orthodox, low burnout).

### Second theme. Coping strategies of mothers of children with special needs

Four strategies emerged from the description of how mothers of children W-SND cope with the difficulties in raising a child W-SND. These encompassed practical, spiritual, social, and emotional coping strategies.

#### Category 1. Practical coping

Mothers with low PB expressed their willingness to receive assistance from their partners or from other close family members in order to provide their children with better care. In comparison, mothers with high PB said they prefer to receive this kind of help only for household tasks, but they avoid asking for help in caring for their children because they feel unable to trust others. In addition, they expressed feelings of guilt due to their perception of caring for the child as their exclusive role. They also noted that they apply to receive financial support for the many treatments their child needs, and sometimes also seek professional treatment for themselves (medical, emotional, psychological):

- “I’m asking my husband and my older daughters for help – mainly with housework, preparing food, tidying up and organizing the house, and buying groceries. I also cannot ask them to take care of the child because he’s a child with many difficulties, including behavioral ones, and it’s hard to look after him and take care of him” (44, ultra-Orthodox, high burnout).
- “I always ask for help, but mostly from my husband and parents. I also sometimes ask them for financial assistance because of the expensive treatments, but when it comes to caring for a child – I’d rather do it alone because I’m her mother and I’ll feel guilty if I do not do it myself and be there for her” (41 non-Orthodox, high burnout).
- “I’m very willing to get help, Both financial help and professional help from a psychologist for myself. I’m proactive all the time. I waited for my son to grow up and be able to express himself, and I have no problem leaving him with a babysitter when I feel like going out with my husband or leaving him with my older children” (47, non-Orthodox, low burnout).

#### Category 2. Spiritual coping

Only ultra-Orthodox mothers with both high and low PB described their faith in God as a source of hope and strength to cope with the difficulties involved in caring for their child. Sometimes, they rely more on their belief in God than in professionals (such as doctors):

- “Believing in God helps me a lot because I understand that everything is in God’s hands and not in the hands of the doctor, and nothing happens to us in life without reason. There’s really no sense of guilt. On the contrary, there is a sense of an anchor, and that the responsibility is on Him (God) because that’s what was chosen, and just as we got a child with problems, miracles are possible, and the child’s situation may get better” (37 ultra-Orthodox, low burnout).
- “I firmly believe that one day it will improve even if the doctors tell us not to, because there is ‘Divine Providence’ and I have a conversation with God and in the end, things will work out” (43, ultra-Orthodox, high burnout).

#### Category 3. Social coping

The mothers said that they receive help and support from the community, neighbors (especially in the ultra-Orthodox community), supportive non-profit organizations and associations, and from the staff of the educational framework, such as the caregivers and kindergarten assistants. Some described that they make sure before seeking childcare help that they can indeed trust the caregiver:

- “I strive to get help only when I trust the relevant person. For example, there are volunteers who want to come and play with my child, but I only allow it if they can handle it. I do not immediately say yes even for trips offered by certain associations. I check and consider whether to send him, and if so, it’s very helpful” (39, non-Orthodox, high burnout).
- “I learned to ask for help and lean on certain people. The staff at my daughter’s kindergarten was very supportive of me and I really feel that they helped me a lot, especially in my coping” (34, non-Orthodox, low burnout).

#### Category 4. Emotional coping

The mothers described a range of emotions that they feel while caring for their child. Mothers with high PB reported both positive and negative emotions. Their most common emotions were pride, joy, sadness, frustration, and anxiety, as compared to all mothers with low PB, who reported mainly positive emotions such as hope, and less negative emotions (except frustration).

- “I am proud of my son’s successes; every time he manages to overcome an obstacle it is our victory, and it makes me happy, all this is accompanied with a lot of stress and anxiety that my child will remain unusual, and that society will discriminate against him. There’s a lot of frustration and helplessness, especially when he has tantrums, and I have to be patient and contain it, but I'm definitely thankful that his situation isn’t worse”)43, ultra-Orthodox, high burnout).
- “I’m very optimistic and happy with this child. I strongly believe in gratitude. I focus on the positive things: how smart and creative and talented he is, and I have no feeling of sadness or fear at all, on the contrary, I have much hope that his communication will improve because he is already constantly improving” (31, ultra-Orthodox, low burnout).

### Third theme. Parental Burnout, consequences, and coping

This theme reflected the implications of PB on mothers of children W-SND and the ways to cope with these implications, in the context of personal and environmental risk factors for PB, alongside personal and environmental protective resources.

#### Category 1. Personal and environmental risk factors

The risk factors for PB and their implications are related both to personal and environmental aspects. Mothers with high PB described these as their actual experience, while mothers with low PB described them according to their perceptions rather than any personal experience:

- “I’m constantly thinking what will happen with her in the future and how it will affect us all. It gives me no rest. I do not sleep at night. It’s really wearing me out and exhausting, especially when a mother is a perfectionist, like me” (41, non-Orthodox, high burnout).
- “When the spouse or family members provide no support; despite the effort and investment, frustration because there is no progress in the child’s condition. The uncertainty regarding what will happen in the future with the child can cause a lot of tension, pressure, thoughts, and burnout, especially emotionally” (34, non-Orthodox, low burnout).

#### Category 2. Personal and environmental protective resources

The interviews revealed recommendations that may serve as protective resources against PB, for example, not to strive for perfection in caring for the child, to know how to ask for help from the environment and the family, to accept reality and to come to terms with it rather than to fight it, to join support groups of mothers with children in a similar situation, to try to concentrate on the positive things of the child, to focus on the present, to make time for themselves (travel, listen to music), and go to work. The spiritual coping strategy, prayer and faith in God, which was described only by mothers from the ultra-Orthodox sector, was found to serve also as a personal protective resource:

- “You must know not to be perfect and not to do everything alone, to let go and know how to get help. Understanding that there is no point in fighting reality and accepting it is better. Believing in God with all my heart, and that one day the child’s condition will improve, because there is Divine Providence. Joining support groups because only people who experience what you experience can let you feel normal. I started working and it helped me a lot, because suddenly I have a normative agenda and it also allows me to get some air” (43 ultra-Orthodox, high burnout).
- “I focus on the positive things that happen to my girl and get strength from them and do not just look at the negative, I really work on myself to know how to ask for and receive help, especially professional help when needed. I also, for example, take time to relax, listen to music, go for walks, and enjoy working out of home as a break from everything involved in caring for my children. I also have a strong, positive and optimistic personality and always look at the half-full glass” (50, non-Orthodox, low burnout).

## Discussion

In this study, 12 mothers of children W-SND from the Ultra-Orthodox and non-Orthodox sectors of Jewish society in Israel who had high or low PB were interviewed. The interviews examined in-depth risk and protective factors for PB, as well as difficulties and coping strategies related to PB. According to the interviews, three themes emerged describing the difficulties mothers face while caring for their child W-SND, their PB, and their coping strategies. In the following sections, a discussion of the findings is presented. At the end of the discussion, Table 2 summarizes the themes and categories, and the primary similarities and differences between ultra-orthodox and non-orthodox mothers of children W-SND with high or low levels of PB. Additionally, the implications for practice are presented.

**Table 2:** Similarities and differences between ultra-Orthodox and non-Orthodox mothers of children W-SND with high or low levels of PB.

	**Similarities**	**Differences**		**Practice**
		Ultra-Orthodox	Non-Orthodox	Implications
*Theme 1. Difficulties involved in the motherhood of children W-SND*				
1. Caregiver burden 2. Task overload	See themselves as the primary caregivers Supervising the child’s functional condition requires time and attention Coordinate treatments	Have more children and therefore more tasks	–––	Development of an intervention program to teach mothers skills for balancing their child’s needs with other tasks
3. Self-neglect (Physical and mental)	*High PB:* Ongoing depression, anxiety, chronic fatigue *Low PB:* Only at certain times do they feel depression, anxiety and fatigue	–––	–––	Identification of mothers’ PB according to self/family report, symptoms of exhaustion, anxiety, depression, self/ environmental neglect
4. Environmental neglect	Neglect couple-hood, other family members and social relationships	Neglection – mainly within the family	Neglection – mainly of social relationships	Increase awareness of family, neighbors, and community regarding ways to help and support these mothers
5. Recognizing the gap between the child W-SND and other same-aged children	Experience difficulty/pain while seeing their child’s development as compared to same-aged children WO-SN	–––	–––	Increase awareness of family, society, and healthcare staff regarding the mother’s need to express authentically her feelings and strengthen her emotional coping abilities
6. Environment’s contradictory expectations from the mother	Expected to feel strong, behave normally, continue functioning although they feel worried and frustrated	Feel anger and pain following others’ expressions toward them (e.g., “God gave you a special child because you are special”)	–––	
*Theme 2. Coping strategies of mothers of children with special needs*				
1. Practical coping	*High PB*: Take care of the child by themselves (due to guilt feelings and lack of trust in others) *Low PB*: Willingness to receive help in childcare from their spouse or close family	–––	–––	Increase awareness regarding ways to provide trustable practical help and support Train students studying related clinical fields as trustworthy professionals for help
2. Spiritual coping		Express faith in God as a source of hope and strength to cope	–––	Exploration of unique cultural coping strategies that may be transferred among societies
3. Social coping	Receive help from supportive non-profit organizations and associations staff of the educational framework	Receive help especially from their community and neighbors	–––	Guidance of the healthcare staff to inquire and offer professional help
4. Emotional coping	*High PB*: Report pride and joy in addition to sadness, frustration, and anxiety *Low PB*: Report mainly positive emotions (hope)	–––	–––	Healthcare staff should pay attention to the mother’s emotional, mental and physical health condition Assure they attend their needed medical treatments
*Theme 3. Parental Burnout, consequences, and coping*				
1. Risk factors: Personal	Pessimistic thoughts Anxiety/constant worries Chronic fatigue Frustration due to lack of progress in the child’s condition, despite efforts	–––	–––	Construction of a cognitive-behavioral workshop to provide coping tools that can be learned
Environmental	Lack of support from family members Difficulty in being able to socialize	–––	–––	Enhance the awareness of family, neighbors and societal-cultural community members, regarding these difficulties
2. Protective resources: Personal	Avoid striving for perfection Accept reality Concentrate on positive aspects of the child Focus on the present	Prayer, faith in God as a divine providence	Travel Listen to music Go out to work	Planning intervention programs that should consider cultural, societal and religious diversity Selection of mentors from the participating mothers themselves
Environmental	Know how to request help Join support groups of mothers with children in a similar situation	–––	–––	Organization of support groups for mothers of children with similar disabilities

### First theme: difficulties involved in the motherhood of children W-SND

According to the mothers, they experience difficulties due to *caregiver burden*, such as the amount of time needed to care for their child and the required constant attention, as well as *task overload*. The latter involves supervising daily tasks adapted to their child’s functional condition and coordinating their many required treatments, especially since the mothers see themselves as their child’s primary caregiver. These findings are supported by the literature which describes the heavy burden that is incurred when a child’s chronic disability affects their daily functioning and creates dependence on the parent, usually the mother as the primary caregiver (Masefield et al., 2020). Regarding the task overload, the literature emphasizes that mothers are required to balance the needs of their child W-SND (treatments, reorganization of the home for accessibility) and those of their other family members and the household (housekeeping, transportation to school and extracurricular activities, going to events) (Pisula, 2011; Masefield et al., 2020).

Additional difficulties mentioned by the mothers were *physical and mental self-neglect* and *neglect of the family and social environment*. Ultra-Orthodox mothers referred mainly to neglect within the family, such as their relationship with their spouse and other children in the family, while non-Orthodox mothers also related to their neglect of social relationships outside the family, such as good friends. These differences may be due to the social norms prevailing in ultra-Orthodox society which view the family institution and its existence as a supreme value (Gonen, 2016). Thus, there is the social and personal expectation to maintain marital and family relationships while simultaneously managing the daily challenges of raising a child W-SND.

Mothers with low PB said that over time they learned ways to adapt to their situation and become less neglectful of themselves and their environment. A possible conclusion from this finding is that a condition for effective coping with the difficulties faced by mothers of a child W-SND is that PB should be treated and reduced. Another difficulty expressed by the interviewed mothers was their *perception of the functional gaps between their children with W-SND and those of other children of the same age*. The literature describes various aspects of the developmental gap resulting from the unique challenges a child W-SND faces. A failure to meet expectations for an improvement in the child’s development exposes the child and the parent to pain, loneliness, and social exclusion (Neely-Barnes et al., 2011; Singal, 2016).

Another difficulty mothers report stems from *conflicting societal expectations about how they should feel or function*. On the one hand, they are expected to be strong, behave normally and continue functioning, while on the other hand, they feel worried and frustrated about the child’s situation. Ultra-Orthodox mothers described many expressions of friends, neighbors, and community members, such as, *“God gave you a special child because you are special.”* They found it difficult to hear these and other similar phrases, which made them feel anger and pain. Similarly, the literature noted the social discourse that reduces the value of those with disabilities, and therefore mothers might feel emotionally overloaded. Mothers may also have a positive attitude and optimism despite their child’s severe disability, which usually is not supported by society (Green, 2007; Broberg, 2011).

### Second theme: coping strategies of mothers of children with special needs

The mothers described four main ways of coping with the difficulties stemming from parenting a child W-SND, i.e., practical, spiritual, social, and emotional coping. Regarding *practical coping strategies*, most mothers with low PB were more willing to receive help for caring for their child from their spouse or close family members, compared to mothers with high PB, who preferred taking care of the child by themselves, mainly due to feeling guilty, the perception that caring for the child is solely their role, and an inability to trust others as caregivers. This is supported by previous studies where the feeling of guilt and exclusive responsibility for the child is explained by the mother’s recognition that only she has the necessary skills and knowledge to manage the child’s condition according to the severity of the disability, and their fear that other caregivers might harm or worsen the child’s clinical condition (Ahmed and Al-Mosawi, 2018; Tsibidaki, 2022). Our findings indicate that mothers’ willingness to accept practical assistance to help with their childcare may contribute to reducing their PB.

Only ultra-Orthodox mothers described *spiritual coping strategies* expressed in their belief in God and prayer that gave them the strength to cope, and a feeling described as an *“anchor”* from a feeling of *“Divine Providence”* leading to a sense of hope. Thus, their perception is that God owns the responsibility (and not only the doctor) and can make miracles and bring about positive change and improvement in their child’s condition. Previous studies have pointed out the strong connections between belief in divine forces and prayer (spiritual-religious coping) as an effective coping strategy that increases mental resilience (Tix and Frazier, 1998; Ahles et al., 2016). Indeed, faith and prayer have served as a positive coping mechanism for people with chronic illness and parents of children W-SND who experience prolonged stress (Tarakeshwar and Pargament, 2001; Tsai et al., 2016). Therefore, we conclude that spiritual religious coping may be an effective resource with PB if it is accepted by the mothers’ societal culture, in this case, the ultra-Orthodox community. However, it is important to note that the relationship between religious beliefs, prayer, and coping is complex, and people who do not hold religious beliefs may use other spiritual coping strategies that are effective for them (e.g., yoga, meditation).

Regarding *social coping strategies*, mothers with high levels of PB prefer to cope with the burden of care on their own because they do not trust others to care for their child, and therefore avoid receiving or seeking social support. This increases the degree of stress and difficulty they experience. This is supported by Vanegas and Abdelrahim (2016) who found that parents who received less support from family and society and had limited means of communication reported more stress than those who received this support. In addition, couples with poor-quality relationships and a lack of support from each other had lower general well-being and reported feeling more frustration and anxiety (Vanegas and Abdelrahim, 2016).

Ultra-Orthodox mothers reported receiving help and support from their community. In line with the literature, the ultra-Orthodox community follows an approach toward people with disabilities in the family that includes, *inter alia*, moral responsibility and kindness toward them (Shaked and Bilu, 2006; Taub and Werner, 2016). However, it was found that some mothers might experience stigma and discrimination by the community, leading to a reluctance to seek help and even social isolation (Mitter et al., 2019).

A unique and interesting contribution of this study which relates to *emotional coping strategies* is that in addition to expressing negative emotions (frustration, sadness, anxiety, and anger) while caring for their child, the mothers also expressed positive emotions, such as pride in the child when he/she succeeds, gratitude for their relative condition, hope for a future improvement, and joy from the child’s positive strengths. However, previous research has focused mainly on negative emotions (e.g., pressure and stress) related to raising a child W-SND (Pillay et al., 2012; Enea and Rusu, 2020). Together with reporting these negative emotions, some studies have described various interventions, such as spirituality and mindfulness, which can reduce stress and enhance resilience and which may arouse positive emotions (e.g., hope) as part of an emotional coping mechanism (Lloyd and Hastings, 2009; Cachia et al., 2016).

### Third theme: parental burnout, consequences, and coping

The previous theme focused on the mothers’ descriptions of the phenomenon of PB, its consequences, and their ways of coping. It should be noted that mothers with a low level of PB described the risk factors they assumed may lead to PB. For example, personal risk factors (such as worrying about the child’s future, and frustration due to no progress in the child’s condition despite efforts) and environmental risk factors (such as lack of support from the family). In contrast, mothers with a high level of PB described actually experiencing the risk factors which they believed led to their PB. For example, they reported having pessimistic thoughts, feeling anxiety regarding their child’s expected future, and suffering from constant worries that deprive them of sleep and causes them chronic fatigue. Other studies conducted among parents of children with chronic diseases have found that parental neuroticism (an imbalance in the emotional state) (Gérain and Zech, 2018; Lebert-Charron et al., 2022) is a personality risk factor for PB. Other studies found additional personal risk factors, such as lack of emotional control (Le Vigouroux and Scola, 2018), perfectionism (Kawamoto et al., 2018; Lebert-Charron et al., 2018), and depression (Roskam et al., 2017; Lebert-Charron et al., 2018). These findings emphasize the unique risk factors of PB found in the current study among mothers of children W-SND.

As a coping resource, mothers with a high level of PB have expressed a strong desire to talk to mothers in a similar situation as an environmental protective resource. According to the literature, parents of children W-SND have unique abilities and skills to help each other and to share and compare their experiences. Hence, they can provide support that cannot be given by others who lack practical experience in coping with PB (Nevin et al., 2022).

Mothers with low PB claimed that keeping optimistic, focusing mainly on the good things (personal), and intentionally asking the environment and society for help (environmental) can significantly reduce their parental stress and thus serve as protective resources against PB. Our study found that these protective resources involved cognitive coping strategies that can be learned (learned resourcefulness) (Rosenbaum, 1990).

Research among parents in general has found that positive thinking and self-compassion related to optimism, and receiving support from the family environment and society, are possible protective resources (Le Vigouroux et al., 2017; Mikolajczak et al., 2018a,b). Therefore, health caregivers may plan programs and workshops to help mothers of children W-SND learn and practice effective coping methods with PB.

Another significant personal protective resource found in the current study was going out to work, which allows mothers to disconnect for a while from caring for their child and his/her dependence on them. Going to work may provide them with a sense of value and routine, social interactions, and financial stability that can help cope with care challenges. As previously described in the literature, a possible explanation is that work may provide a break from the demands associated with caring for a child and provide a sense of accomplishment and independence leading to positive well-being and satisfaction in life (Ginevra et al., 2018).

Finally, mothers from the ultra-Orthodox sector, regardless of their level of burnout, described a unique personal strategy for coping with PB through their belief in God, whom they perceive as a significant positive resource that protects them from PB. Faith influences how the child’s disability is perceived since ultra-Orthodox Judaism dictates an unquestioning belief in the Divine Providence which gave life to the child. Similarly, it was found that caring for a child W-SND is seen by ultra-Orthodox parents as their mission that demands strength and tools to cope and which they believe will be provided by God (Kandel, 2010).

## Conclusions and practice implications

The strong sense of caregiver burden placed on mothers of children W-SND as primary caregivers is a heavy weight of responsibility. This ongoing situation may lead to PB, including physical and emotional exhaustion, self-neglect, and neglect of the family and social environment. Moreover, this state is sometimes accompanied by feelings of guilt which prevents mothers from receiving practical and social assistance and support in caring for their child. It also might create difficulties in developing coping strategies and utilizing efficient techniques.

Therefore, it is important to help mothers of children W-SND cope with their difficulties, risk factors and consequences of PB and provide accessible coping methods and protective resources. To meet these aims, health staff, community leaders and members, neighbors and family members should be guided to increase awareness regarding providing trustable practical help and support to mothers of children W-SND.

Therefore, possible implications for practice are:

- Healthcare staff who have frequent contact with the mothers should identify the level of PB of mothers of children W-SND according to the mother’s or her family’s report. They should also pay attention to mothers’ symptoms of exhaustion, anxiety, depression, self-neglect (e.g., avoiding eating, deprivation of sleep), and environmental neglect (e.g., loneliness, avoidance of social company), to assure that they attend to their own needed medical treatments.
- Healthcare staff should be guided to inquire and offer professional help (in addition to possible existing support of family and friends).
- Therapeutic teams in the health system could collaborate with the local authorities and the community to organize support groups for mothers of children with similar disabilities, as a protective resource that enables coping with PB.
- The mother’s spouse, family members, society, community, and healthcare staff should be aware of the mother’s need to express her feelings authentically and strengthen her emotional coping abilities.
- An intervention program should be planned for training and learning skills that can assist mothers in regulating their load of tasks (for example, prioritizing tasks, delegating responsibilities, and developing an understanding that the responsibility for the child W-SND and the entire family does not lie solely on the mother’s shoulders), in order to balance their child’s needs with their own personal needs.
- This intervention program should consider cultural, societal, and religious diversity among mothers. It was found that ultra-Orthodox mothers describe belief and prayer as a religious spiritual unique coping strategy that strengthens them and gives them hope. This unique coping strategy may be transferred among societies.
- Mentors of these programs may be selected from the participating mothers themselves (as they can identify with the experience of motherhood of a child W-SND based on their own experience).
- A dedicated program should be developed and operated for training students studying clinical fields related to the treatment of children W-SND (nursing, occupational therapists, physical-therapists, special education teachers). These students may serve as a pool of trustable professional help for the mothers and may be rewarded by scholarships.
- A cognitive-behavioral workshop should be initiated to assist mothers in coping with negative thoughts, worries and a sense of burden due to the lack of sufficient support. The workshop will provide coping tools and skills that mothers can learn to help themselves emotionally and positively impact their behavior (learned resourcefulness, 57).

These suggested intervention programs should operate nationally and be suited to multiple cultures. The programs should be carried out by qualified health caregivers and followed by research and evaluation.

### Limitations

The sample included only Jewish mothers; in future studies, it is recommended to add other cultural, religious, and ethnic groups to represent additional social and cultural societies in the population that may show different risk factors for PB. Moreover, the current study was conducted among mothers of children W-SND only, as they are considered the ‘main informal caregivers’ in non-Orthodox and ultra-Orthodox societies (Kandel, 2010; Masefield et al., 2020). Although the sample size is sufficient to achieve saturation in the analysis of the qualitative data, it is relatively small. Thus, a future study on a larger sample is recommended. Due to the increasing involvement of fathers in the care of children in some sectors and classes in Israeli society (Cahaner and Malach, 2021), it is recommended to conduct a similar study among fathers of children with special needs and also among both parents. Expanding the sample from mothers only to both parents may contribute to the planning and development of intervention programs for the whole family. It should be borne in mind that the Jewish ultra-Orthodox religion tends to conceive disabilities as God’s will. Therefore, a main difference exists between ultra-Orthodox and non-Orthodox mothers (Kandel, 2010). Finally, social desirability might have affected the mothers’ answers (Vesely and Klöckner, 2020) e.g., avoidance of reporting difficulties that may be perceived as weaknesses, or that might threaten the family’s status in their community.

## Data availability statement

The raw data supporting the conclusions of this article will be made available by the authors, without undue reservation.

## Ethics statement

The study was approved by the Helsinki Committee of Meuhedet Health Service Clinics (Approval number 02290120) and by Tel-Aviv University’s ethics committee (Approval number 0001222–2). The studies were conducted in accordance with the local legislation and institutional requirements. The participants provided their written informed consent to participate in this study.

## Author contributions

YF: Conceptualization, Investigation, Methodology, Validation, Writing – review & editing, Data curation, Formal analysis, Funding acquisition, Resources, Software, Writing – original draft. SB: Conceptualization, Investigation, Methodology, Validation, Writing – review & editing, Project administration, Supervision, Visualization. MI: Conceptualization, Investigation, Methodology, Project administration, Supervision, Validation, Visualization, Writing – review & editing.

## References

[ref1] Ahles J. J. Mezulis A. H. Hudson M. R. (2016). Religious coping as a moderator of the relationship between stress and depressive symptoms. Psychol. Relig. Spiritual. 8, 228–234. doi: 10.1037/rel0000039

[ref2] Ahmed E. A. Al-Mosawi K. M. (2018). Family stress and its coping strategies related to children with cerebral palsy at azadi teaching hospital in Kirkuk City. Ind. J. Public Health Res. Dev. 9, 1286–1291. doi: 10.5958/0976-5506.2018.00908.7

[ref3] Braun V. Clarke V. (2019). To saturate or not to saturate? Questioning data saturation as a useful concept for thematic analysis and sample-size rationales. Qual. Res. Sport Exerc. Health 13, 201–216. doi: 10.1080/2159676X.2019.1704846

[ref4] Bristow S. Jackson D. Shields L. Usher K. (2018). The rural mother's experience of caring for a child with a chronic health condition: an integrative review. J. Clin. Nurs. 27, 2558–2568. doi: 10.1111/jocn.14360, PMID: 29575208

[ref5] Broberg M. (2011). Expectations of and reactions to disability and normality experienced by parents of children with intellectual disability in Sweden. Childcare Health Dev. 37, 410–417. doi: 10.1111/j.1365-2214.2010.01198.x, PMID: 21276035

[ref6] Cachia R. L. Anderson A. Moore D. W. (2016). Mindfulness, stress and well-being in parents of children with autism spectrum disorder: a systematic review. J. Child Fam. Stud. 25, 1–14. doi: 10.1007/s10826-015-0193-8

[ref7] Cahaner L. Malach G. Shnaton hahevra haOrthodoxt be'Israel [the statistical report on ultra-orthodox (orthodox) sector in Israel]. Jerusalem: The Israeli institute of Democracy. (2021). Available at: [Hebrew https://en.idi.org.il/media/17712/final-idi_research_haredim_web.pdf]

[ref8] Carroll D. W. Families of children with developmental disabilities: Understanding stress and opportunities for growth. New York: American Psychology Association (2013).

[ref9] Constantinou C. S. Georgiou M. Perdikogianni M. (2017). A comparative method for themes saturation (CoMeTS) in qualitative interviews. Qual. Res. 17, 571–588. doi: 10.1177/1468794116686650

[ref10] Creswell JW Poth CN. Qualitative inquiry and research design: Choosing among five approaches. London: Sage (2018).

[ref11] Enea V. Rusu D. M. (2020). Raising a child with autism spectrum disorder: a systematic review of the literature investigating parenting stress. J. Ment. Health Res. Intellect. Disabil. 13, 283–321. doi: 10.1080/19315864.2020.1822962

[ref12] Findling Y. Barnoy S. Itzhaki M. (2022). Burden of treatment, emotion work and PB of mothers to children with or without special needs: a pilot study. Curr. Psychol. 42, 19273–19285. doi: 10.1007/s12144-022-03074-2

[ref13] Fram S. M. (2013). The constant comparative analysis method outside of grounded theory. Qual. Rep. 18, 1–25. doi: 10.46743/2160-3715/2013.1569

[ref14] Fugard A. J. Potts H. W. (2015). Supporting thinking on sample sizes for thematic analyses: a quantitative tool. Int. J. Soc. Res. Methodol. 18, 669–684. doi: 10.1080/13645579.2015.1005453

[ref15] Fusch P. Fusch G. E. Ness L. R. (2018). Denzin’s paradigm shift: revisiting triangulation in qualitative research. J. Sustain. Soc. Change 10, 19–32. doi: 10.5590/JOSC.2018.10.1.02

[ref16] Gérain P. Zech E. (2018). Does informal caregiving lead to PB? Comparing parents having (or not) children with mental and physical issues. Front. Psychol. 9:884. doi: 10.3389/fpsyg.2018.00884, PMID: 29928242 PMC5997813

[ref17] Ginevra M. C. Di Maggio I. Santilli S. Sgaramella T. M. Nota L. Soresi S. (2018). Career adaptability, resilience, and life satisfaction: a mediational analysis in a sample of parents of children with mild intellectual disability. J. Intellect. Develop. Disabil. 43, 473–482. doi: 10.3109/13668250.2017.1293236

[ref18] Glaser B. G. Strauss A. L. (1967). Discovery of grounded theory: Strategies for qualitative research. New York: Aldine.

[ref19] Gonen P, Ultra-orthodox women. The center for the study of the ultra-orthodox society. Policy paper number 462. Jerusalem Institute for Policy Studies (2016). Available at: [Hebrew https://jerusaleminstitute.org.il/wp-content/uploads/2019/05/PUB_נשים-חרדיות.pdf]

[ref20] Green S. E. (2007). "We’re tired, not sad": benefits and burdens of mothering a child with a disability. Soc. Sci. Med. 64, 150–163. doi: 10.1016/j.socscimed.2006.08.025 17014946

[ref21] Ivry T. Teman E. Frumkin A. (2011). God-sent ordeals and their discontents: ultra-orthodox Jewish women negotiate prenatal testing. Soc. Sci. Med. 72, 1527–1533. doi: 10.1016/j.socscimed.2011.03.007, PMID: 21470732

[ref22] Kalaji T. Brown-Levinson A. Differentiated integration: Ultra-orthodox academics in the Israeli economy, policy research 115. Hebrew: Israel Democracy Institute. (2017).

[ref23] Kandel I. (2010). “The attitudes of the ultra-orthodox sector towards different children” in And your brother lives with you: The attitude towards people with unique needs and their integration into society. ed. Rahimi M. (Rehovot: Orot Israel College)

[ref24] Kawamoto T. K. Furutani K. Alimardani M. (2018). Preliminary validation of Japanese version of the PB inventory and its relationship with perfectionism. Front. Psychol. 9:970. doi: 10.3389/fpsyg.2018.00970, PMID: 29973893 PMC6019475

[ref25] Kegler M. C. Raskind I. G. Comeau D. L. Griffith D. M. Cooper H. L. Shelton R. C. (2019). Study design and use of inquiry frameworks in qualitative research. Health Educ. Behav. 46, 24–31. doi: 10.1177/10901981187950, PMID: 30227081 PMC6386610

[ref26] Le Vigouroux S. L. Scola C. (2018). Differences in parental burnout: influence of demographic factors and personality of parents and children. Front. Psychol. 9:887. doi: 10.3389/fpsyg.2018.00887, PMID: 30013491 PMC6036141

[ref27] Le Vigouroux S. Scola C. Raes M. E. Mikolajczak M. Roskam I. (2017). The big five personality traits and PB: protective and risk factors. Personal. Individ. Differ. 119, 216–219. doi: 10.1016/j.paid.2017.07.023

[ref28] Lebert-Charron A. Dorard G. Boujut E. Wendland J. (2018). Maternal burnout syndrome: contextual and psychological associated factors. Front. Psychol. 9:885. doi: 10.3389/fpsyg.2018.00885, PMID: 29922203 PMC5996184

[ref29] Lebert-Charron A. Wendland J. Vivier-Prioul S. Boujut E. Dorard G. (2022). Does perceived partner support have an impact on mothers’ mental health and PB? Marriage Fam. Rev. 58, 362–382. doi: 10.1080/01494929.2021.1986766

[ref30] Lincoln YS Guba EG. Naturalistic inquiry. London: Sage (1985).

[ref31] Lindström C. Åman J. Lindahl Norberg A. (2010). Increased prevalence of burnout symptoms in parents of chronically ill children. Acta Paediatr. 99, 427–432. doi: 10.1111/j.1651-2227.2009.01586.x, PMID: 19912139

[ref32] Lindström C. Åman J. Norberg A. L. (2011). Parental burnout in relation to sociodemographic, psychosocial and personality factors as well as disease duration and glycaemic control in children with type 1 diabetes mellitus. Acta Paediatr. 100, 1011–1017. doi: 10.1111/j.1651-2227.2011.02198.x, PMID: 21414025

[ref33] Liu Z. Heffernan C. Tan J. (2020). Caregiver burden: a concept analysis. Int. J. Nurs. Sci. 7, 438–445. doi: 10.1016/j.ijnss.2020.07.012, PMID: 33195757 PMC7644552

[ref34] Lloyd T. J. Hastings R. (2009). Hope as a psychological resilience factor in mothers and fathers of children with intellectual disabilities. J. Intellect. Disabil. Res. 53, 957–968. doi: 10.1111/j.1365-2788.2009.01206.x, PMID: 19744261

[ref35] Masefield S. C. Prady S. L. Sheldon T. A. Small N. Jarvis S. Pickett K. E. (2020). The caregiver health effects of caring for young children with developmental disabilities: a meta-analysis. Matern. Child Health J. 24, 561–574. doi: 10.1007/s10995-020-02896-5, PMID: 32048172 PMC7170980

[ref36] Mikolajczak M. Brianda M. E. Avalosse H. Roskam I. (2018a). Consequences of PB: its specific effect on child neglect and violence. Child Abuse Negl. 80, 134–145. doi: 10.1016/j.chiabu.2018.03.025 29604504

[ref37] Mikolajczak M. Raes M. E. Avalosse H. Roskam I. (2018b). Exhausted parents: sociodemographic, child-related, parent-related, parenting and family-functioning correlates of PB. J. Child Fam. Stud. 27, 602–614. doi: 10.1007/s10826-017-0892-4

[ref38] Mitter N. Ali A. Scior K. (2019). Stigma experienced by families of individuals with intellectual disabilities and autism: a systematic review. Res. Dev. Disabil. 89, 10–21. doi: 10.1016/j.ridd.2019.03.001, PMID: 30875608

[ref39] Neely-Barnes S. L. Hall H. R. Roberts R. J. Graff J. C. (2011). Parenting a child with an autism spectrum disorder: public perceptions and parental conceptualizations. J. Fam. Soc. Work. 14, 208–225. doi: 10.1080/10522158.2011.571539

[ref40] Nelson S. K. Kushlev K. Lyubomirsky S. (2014). The pains and pleasures of parenting: when, why, and how is parenthood associated with more or less well-being? Psychol. Bull. 140, 846–895. doi: 10.1037/a0035444 24491021

[ref41] Nevin S. M. Wakefield C. E. Dadich A. Le Marne F. Macintosh R. Beavis E.. (2022). Hearing parents' voices: a priority-setting workshop to inform a suite of psychological resources for parents of children with rare genetic epilepsies. PEC Innov. 1:100014. doi: 10.1016/j.pecinn.2021.100014, PMID: 37364015 PMC10194388

[ref42] Noble H. Heale R. (2019). Triangulation in research, with examples. Evid. Based Nurs. 22, 67–68. doi: 10.1136/ebnurs-2019-103145 31201209

[ref43] Nomaguchi K. Milkie M. A. (2020). Parenthood and well-being: a decade in review. J. Marriage Fam. 82, 198–223. doi: 10.1111/jomf.12646, PMID: 32606480 PMC7326370

[ref44] Pillay D. Girdler S. Collins M. Leonard H. (2012). "It’s not what you were expecting, but it’s still a beautiful journey": the experience of mothers of children with down syndrome. Disabil. Rehabil. 34, 1501–1510. doi: 10.3109/09638288.2011.650313 22324752

[ref45] Pisula E. (2011). “Parenting stress in mothers and fathers of children with autism spectrum disorders” in A comprehensive book on autism Spectrum disorders. ed. Mohammadi M. R.

[ref46] Rosenbaum M. (1990). “The role of learned resourcefulness in the self-control of health behavior” in Learned resourcefulness: On coping skills, self-control, and adaptive behavior. ed. Rosenbaum M.

[ref47] Roskam I. Brianda M. E. Mikolajczak M. A. (2018). Step forward in the conceptualization and measurement of parental burnout: the parental burnout assessment (PBA). Front. Psychol. 9:758. doi: 10.3389/fpsyg.2018.00758, PMID: 29928239 PMC5998056

[ref48] Roskam I. Raes M. E. Mikolajczak M. (2017). Exhausted parents: development and preliminary validation of the parental burnout inventory. Front. Psychol. 8;163. doi: 10.3389/fpsyg.2017.00163 28232811 PMC5298986

[ref49] Shaked M. Bilu Y. (2006). Grappling with affliction: autism in the Jewish ultra-orthodox community in Israel. Cult. Med. Psychiatry 30, 1–27. doi: 10.1007/s11013-006-9006-2, PMID: 16783528

[ref50] Singal N. (2016). Schooling children with disabilities: parental perceptions and experiences. Int. J. Educ. Dev. 50, 33–40. doi: 10.1016/j.ijedudev.2016.05.010

[ref51] Tarakeshwar N. Pargament K. I. (2001). Religious coping in families of children with autism. Focus Autis. Other Dev. Disabil. 16, 247–260. doi: 10.1177/108835760101600408

[ref52] Taub T. Werner S. (2016). What support resources contribute to family quality of life among religious and secular Jewish families of children with developmental disability? J. Intellect. Develop. Disabil. 41, 348–359. doi: 10.3109/13668250.2016.1228859

[ref53] Tix A. P. Frazier P. A. (1998). The use of religious coping during stressful life events: Main effects, moderation, and mediation. J. Consult. Clin. Psychol. 66, 411–422. doi: 10.1037/0022-006X.66.2.411, PMID: 9583344

[ref54] Tong A. Sainsbury P. Craig J. (2007). Consolidated criteria for reporting qualitative research (COREQ): a 32-item checklist for interviews and focus groups. Int. J. Qual. Health Care 19, 349–357. doi: 10.1093/intqhc/mzm042, PMID: 17872937

[ref55] Tsai T. J. Chung U. L. Chang C. J. Wang H. H. (2016). Influence of religious beliefs on the health of cancer patients. Asian Pac. J. Cancer Prev. 17, 2315–2320. doi: 10.7314/APJCP.2016.17.4.2315 27221937

[ref56] Tsibidaki A. (2022). Parental perceptions of cerebral palsy and expectations of the operation outcomes in Greece and in Italy. Fam. J. 30, 630–637. doi: 10.1177/10664807221079284

[ref57] Van Bakel H. J. Van Engen M. L. Peters P. (2018). Validity of the PB inventory among Dutch employees. Front. Psychol. 9:697. doi: 10.3389/fpsyg.2018.00697 29875711 PMC5974116

[ref58] Vanegas S. B. Abdelrahim R. (2016). Characterizing the systems of support for families of children with disabilities: a review of the literature. J. Fam. Soc. Work. 19, 286–327. doi: 10.1080/10522158.2016.1218399

[ref59] Vesely S. Klöckner C. A. (2020). Social desirability in environmental psychology research: three meta-analyses. Front. Psychol. 11:1395. doi: 10.3389/fpsyg.2020.01395, PMID: 32793022 PMC7393925

[ref60] Vinayak S. Dhanoa S. (2017). Relationship of PB with parental stress and personality among parents of neonates with hyperbilirubinemia. Int. J. Indian Psychol. 4, 102–111. doi: 10.25215/0402.112

